# A Case of ST‐Elevation Acute Myocardial Infarction in a Nonhospitalized Patient with SARS‐CoV‐2 Pneumonia: Treatment with Primary Percutaneous Coronary Intervention

**DOI:** 10.1002/ccr3.4785

**Published:** 2021-09-15

**Authors:** Ryosuke Shimai, Shohei Ouchi, Tetsuro Miyazaki, Koji Hirabayashi, Hiroshi Abe, Kosuke Yabe, Masaaki Maki, Hiroyuki Isogai, Takeshi Wada, Dai Ozaki, Fuminori Odagiri, Makoto Hiki, Kenji Yaginuma, Ken Yokoyama, Takashi Tokano, Tohru Minamino

**Affiliations:** ^1^ Department of Cardiology Juntendo Urayasu Hospital Urayasu Japan; ^2^ Department of Cardiovascular Biology and Medicine Juntendo University School of Medicine Bunkyo‐ku Japan

**Keywords:** coagulopathy, COVID‐19, infection prevention protocol, out‐of‐hospital onset, ST‐elevation myocardial infarction

## Abstract

We experienced a case of primary percutaneous coronary intervention for ST‐elevation myocardial infarction (STEMI) with coronavirus disease 2019 (COVID‐19) using appropriate infection prevention protocol. However, recanalization was difficult due to severe coagulopathy. Further researches are needed to clarify optimal treatment for STEMI in patients with COVID‐19.

## Graphical abstract

To perform primary PCI for patients with concomitant acute coronary syndrome and COVID‐19 as soon as possible, an appropriate infection prevention protocol in place should be prepared.

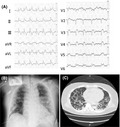

## 1 INTRODUCTION

Coronavirus disease 2019 (COVID‐19) is an infectious disease caused by severe acute respiratory syndrome coronavirus 2 (SARS‐CoV‐2). The rapid spread of this disease worldwide led to an ongoing pandemic. Although the respiratory problems caused by this disease have drawn much attention, diverse cardiovascular symptoms, including acute coronary syndrome, myocarditis, pericarditis, and pericardial effusion,^1^ have also been associated with COVID‐19. For cases of ST‐elevation myocardial infarction (STEMI) that developed during hospitalization for COVID‐19, treatment with percutaneous coronary intervention (PCI) has been reported.^2^, ^3^, ^4^ We report PCI treatment of a case of acute myocardial infarction that occurred out of a hospital in a patient with COVID‐19.

### 1.1 Case Presentation

A 66‐year‐old man (body height, 168 cm; body weight, 88.5 kg) experienced dyspnea, a sensation of chest tightness, and back pain for a few days before he was brought to our hospital on an emergency basis. His symptoms became aggravated suddenly as he was about to get off a taxi on his way home from work. He presented with a history of close contact with a coworker with COVID‐19 and was waiting for instructions from a public health center.

**FIGURE 1:**
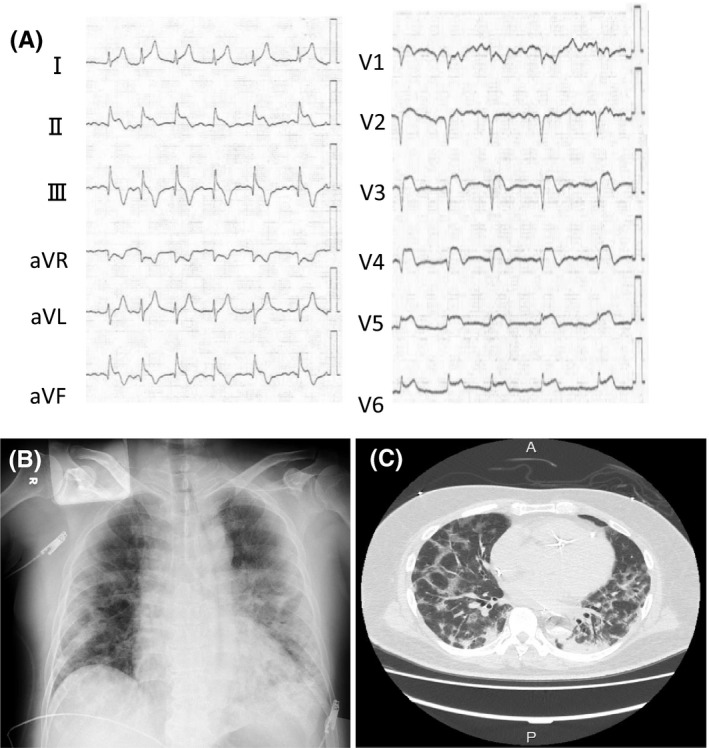
Twelve‐lead electrocardiography on admission revealed atrial fibrillation; ST‐elevation in the II, III, aVF, and V2‐6 leads; and ST depression in aVR leads (A). Chest radiography on admission showed reduced radiolucency in both lung fields (B). Chest computed tomography after primary percutaneous coronary intervention (PCI) showed ground‐glass opacities distributed unevenly in both lungs, as well as left‐sided pleural effusion (C).

In the ambulance, the following conditions were documented: Glasgow Coma Scale score, E4V5M6; heart rate, 80/min and irregular; blood pressure, 100/60 mmHg; body temperature, 38.4ºC; oxygen saturation, 98% (O_2_ delivered with reservoir mask at a rate of 15 L/min). On admission, his white blood cell count was 22,300/µL; hemoglobin, 14.7 g/dL; platelet count, 35 × 10^4^/µL; creatinine level, 2.23 mg/dL; creatine kinase level, 2582 IU/L; creatine kinase myocardial band level, 164 ng/mL; low‐density lipoprotein cholesterol level, 97 mg/dL; high‐density‐lipoprotein cholesterol level, 19 mg/dL; triglyceride level, 173 mg/dL; hemoglobin A1c level, 7.9%; C‐reactive protein level, 13.8 mg/dL; troponin T level, 9.640 ng/mL; *N*‐terminal pro‐brain natriuretic peptide level, 11753 pg/mL; procalcitonin level, 0.62 ng/mL; d‐dimer level, 16.34 µg/mL; and soluble fibrin level, 113.1 µg/mL. An electrocardiogram showed atrial fibrillation; ST‐elevation in II, III, aVF, and V2–V6 leads; and ST depression in aVR leads (Figure 1A). Echocardiography revealed severe hypokinesis extending from the anterior wall to the apex and the inferior wall in left ventricle, with an approximate ejection fraction of 20%. We diagnosed STEMI and decided to perform emergency coronary angiography (CAG). A chest radiograph showed infiltrative shadows in both lungs and tracheal intubation was performed in the emergency outpatient unit (Figure 1B). Continuous infusion of noradrenaline (0.1 μg/kg/min) was started because his systolic blood pressure dropped below 80 mmHg. After heparin (5,000 U) was administered intravenously, the patient was transferred to the cardiac catheterization room.

**FIGURE 2:**
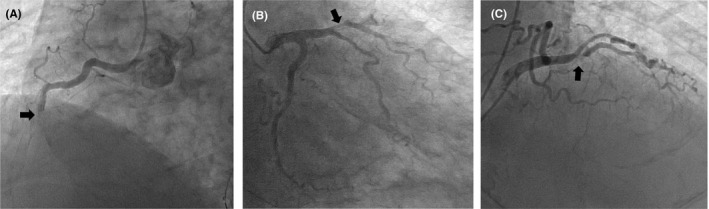
Coronary angiography showed total occlusion at the middle level of the right coronary artery (A; 50° left anterior oblique view) and at the middle level of the left anterior descending artery (B: 30° right anterior oblique (RAO) view and 30° caudal view; C: 20° RAO view and 40° caudal view).

**FIGURE 3:**
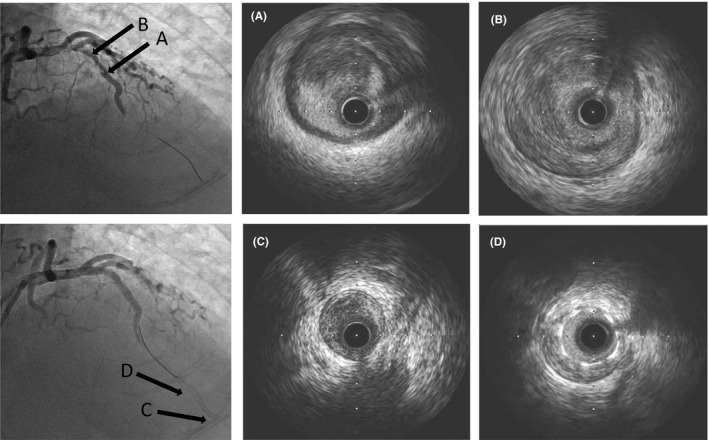
After thrombus aspiration, no translucencies were observed in the lesion or in distal blood flow (upper row). Initial intravascular ultrasonography (IVUS) showed a low‐density area suspected of representing thrombi in the 12 o'clock direction (A) and a large amount of plaque in the proximal site (B). Final angiography showed no distal blood flow (lower row). Final IVUS showed thrombi and plaque at the distal end of the stent (C) and no intrastent blood flow (D).

CAG was performed by cardiologists, emergency physicians, clinical engineers, nurses, and radiological technologists who donned appropriate personal protective equipment before entering the room. CAG revealed total occlusion of the middle of the right coronary artery (RCA) and left anterior descending artery (LAD; Figure 2). After a loading dose of dual antiplatelet therapy (aspirin, 162 mg; clopidogrel, 300 mg), an intra‐aortic balloon pump catheter was inserted, and a 7‐Fr guiding catheter was inserted into the left coronary artery. We removed white material, which did not at a first glance appear to be a typical thrombus, with an aspiration catheter, but blood flow did not improve. The onset‐to‐device time and door‐to‐device time were 116 and 84 minutes, respectively. Subsequent intravascular ultrasonography (IVUS) revealed several structures, which were thought to be thrombi; therefore, we performed plain old balloon angioplasty (Figure 3A, 3B). After the balloon was expanded, blood flow improved slightly in the LAD but not distally. Next, we placed an everolimus‐eluting stent (4.0 × 38 mm) in the middle of the LAD, but peripheral blood flow was still not observed. Repeated IVUS revealed severe stenosis caused by plaque with thrombi. An additional balloon was inflated at the distal site, and a cutting balloon was also used. Another everolimus‐eluting stent (2.25 × 38 mm) was placed at the distal site, but no blood flow improvements were seen angiographically.

We moved a double‐lumen catheter to the distal LAD and performed CAG from the distal LAD, which revealed retrograde blood flow up to the middle of the LAD and collateral circulation from the septal branch to the RCA. Repeated IVUS examination revealed thrombi and plaque at the distal end of the stent and no intrastent blood flow (Figure 3C, 3D). Then, we tried to perform PCI for the total occlusion of RCA, but it was unsuccessful. At this point, 7 hours passed since the patient entered the cardiac catheterization room, and so we finished the procedure. The levels of activated clotting time during PCI were maintained at 280–310 seconds. After PCI, pathological examination proved the aspirated white material was white thrombi. Chest computed tomography performed after PCI showed ground‐glass opacities distributed unevenly in both the lungs, as well as left‐sided pleural effusion (Figure 1C). After the patient was transferred to the intensive care unit, he showed pulseless electrical activity, and cardiopulmonary resuscitation (CPR) was initiated. Return of spontaneous circulation was achieved with 1 mg of epinephrine and one cycle of CPR. Veno‐arterial extracorporeal membrane oxygenation was considered, but his family decided against it. The blood pressure was maintained through the continuous administration of noradrenaline, dobutamine, and adrenaline. His nasopharyngeal swab was tested with polymerase chain reaction for SARS‐CoV‐2, and COVID‐19‐related pneumonia was diagnosed. Remdesivir treatment was initiated at a dose of 200 mg/day on the 5th day of hospitalization and continued at 100 mg/day. Because of the possibility of concomitant bacterial pneumonia, ampicillin sodium/sulbactam sodium (9 g/day) was also administered. However, the patient's oxygenation level worsened, and he died on the 7th day. It was difficult to confirm a main cause of death because we were not permitted to dissect patients due to COVID‐19. We assumed that he had died from both cardiogenic shock due to myocardial infarction and septic shock due to covid‐19 infection.

## 2 DISCUSSION

The cardiovascular symptoms of COVID‐19 are diverse; they include myocardial infarction, myocarditis, nonischemic cardiomyopathy, coronary spasm, pericarditis, and Takotsubo cardiomyopathy. Elevated levels of cardiac biomarkers suggest a poor prognosis.^1^ In early clinical experience in Wuhan, China, where the first cases of COVID‐19 were identified, cardiac muscle damage was found in approximately 27.8% (52/187) of patients and was associated with elevated levels of cardiac troponin T.^5^ The exact mechanism underlying the development of cardiac muscle damage in COVID‐19 remains unclear; however, some plausible mechanisms that have been proposed include plaque rupture, cytokine storm, excessive hypoxia, systemic inflammatory response, coronary spasm, microthrombosis, or microangiopathy resulting from disordered coagulation, and direct injuries to the vascular endothelium.^6^

Amid the ongoing COVID‐19 pandemic, management of patients with concomitant acute coronary syndrome and COVID‐19 is becoming difficult. After diagnosis of COVID‐19, primary PCI may be significantly delayed in affected patients, although prompt primary PCI is the standard treatment for STEMI. In addition, asymptomatic or minimally symptomatic cases of COVID‐19 may not be identified through polymerase chain reaction methods alone^7^; therefore, this difficult situation can occur even in patients with undiagnosed COVID‐19. To perform primary PCI promptly and safely for patients with confirmed or suspected COVID‐19, both appropriate personal protective equipment and a dedicated cardiac catheterization room should be available.^1^ For our patient, we were able to perform PCI on an emergency basis because the patient was suspected to present with COVID‐19 before arrival, and our hospital had an infection prevention protocol in place. In brief, primary PCI for STEMI should be considered using appropriate personal protective equipment in severe COVID‐19 cases, and endotracheal intubation should be performed prior to PCI if respiratory status is unstable. Even in suspected cases of COVID‐19, primary PCI should be considered, and endotracheal intubation should be performed before primary PCI if respiratory status is unstable. In very severe cases of COVID‐19; however, conservative treatment should be chosen without invasive treatment including catheterization. In our protocol, prompt reperfusion therapy should be considered rather than chest CT, thus chest CT is performed after PCI. In addition, positive room pressure of the cardiac catheterization room should be turned off. Although treatment was concluded without achieving revascularization, the door‐to‐device time was within 90 minutes, and thus, primary PCI was performed as quickly as in other cases of STEMI.

In our patient, two arteries were totally occluded. Presumably, the RCA had been chronically occluded, a collateral artery from the LAD to the RCA had consequently formed, and the STEMI was triggered by acute occlusion of the LAD. Revascularization was difficult because of peripheral thromboembolism, and the levels of soluble fibrin and d‐dimer remained remarkably high after his arrival at the hospital. Levels of thrombosis markers are commonly elevated even in non–COVID‐19‐related cases of acute myocardial infarction.^8^ However, soluble fibrin and d‐dimer levels in this case were remarkably high and remained so during the continuous administration of heparin. In severe cases of COVID‐19, coagulopathy has been noted, and disseminated intravascular coagulation (DIC)‐like massive intravascular clot formation is common.^2^ In comparison with bacterial sepsis‐associated coagulopathy/DIC, prolonged prothrombin time, prolonged activated partial thromboplastin time, and decreased antithrombin activity are infrequent with COVID‐19, and thrombocytopenia is relatively rare. The mechanism of coagulopathy by COVID‐19 has not been explained fully. The involvement of disordered immune responses with regard to inflammatory cytokines, lymphocyte death, hypoxia, endothelial damage, and other causes has been speculated.^9^ Higher serum levels of D‐dimer, which is produced after activation of coagulation and fibrinolysis, were associated with a poor prognosis in patients with COVID‐19. Several studies have demonstrated D‐dimer exceeding 3.0 μg/ml to be a cut‐off value for diagnosing patients with DIC and guiding the anticoagulation treatment to decrease mortality rate in patients with COVID‐19.^10^ For patients with notably high D‐dimer levels as in the present case, administration of a thrombolytic agent might be considered in addition to PCI, although the optimal treatment for acute coronary syndrome in patients with COVID‐19 is not yet established because of insufficient experience.

## 3 CONCLUSION

We described a case in which primary PCI was performed for STEMI that occurred in an unhospitalized patient with undiagnosed COVID‐19. Neither COVID‐19 nor SARS‐CoV‐2 is expected to be eradicated in the near future. Because more cases similar to ours are likely to occur, an appropriate infection prevention protocol should be prepared in each healthcare facility. In addition, the optimal treatment for acute coronary syndrome in patients with COVID‐19 and remarkable coagulopathy is not established because of insufficient experience. Further research is warranted to clarify appropriate and rational therapeutic approaches.

## 4 FUNDING STATEMENT

None declared.

## 5 ETHICS STATEMENT

Patient's family verbal consent had been obtained to use the material.

## CONFLICT OF INTEREST

None declared.

## AUTHORS CONTRIBUTIONS

Ryosuke Shimai and Tetsuro Miyazaki: Brief summary of contribution. Shohei Ouchi: Conceptualization of the presented idea. Koji Hirabayashi, Hiroshi Abe, Kosuke Yabe, Masaaki Maki, Hiroyuki Isogai, and Fuminori Odagiri: Data collection. Takeshi Wada and Dai Ozaki: Data collection and supervised the findings of this work. Makoto Hiki, Kenji Yaginuma, Ken Yokoyama, Takashi Tokano, and Tohru Minamino: Supervised the findings of this work.
